# Significant enhancement of the bias stability of Zn-O-N thin-film transistors via Si doping

**DOI:** 10.1038/s41598-020-57642-2

**Published:** 2020-01-20

**Authors:** Aeran Song, Hyun-Woo Park, Hyoung-Do Kim, Hyun-Suk Kim, Kwun-Bum Chung

**Affiliations:** 1 Division of Physics and Semiconductor Science, Dongguk University, Seoul, 04620 Republic of Korea; 2 Department of Materials Science and Engineering, Chungnam National University, Daejeon, 34134 Republic of Korea

**Keywords:** Electronic devices, Electronic devices

## Abstract

Si doping was used to significantly improve the bias stability of ZnON thin-film transistors. Si 3 W (~1%) doped ZnON TFTs showed a saturation mobility of 19.70 cm^2^/Vs along with remarkable improvements in the threshold voltage shift for negative gate bias stress (NBS) within 1.69 V. The effects of Si doping were interpreted by the experimental correlation between device performance and physical analysis, as well as by the theoretical calculation. Si doping induces the reduction of N-related defects by increasing stoichiometric Zn_3_N_2_, and decreasing nonstoichiometric Zn_x_N_y_. In addition, Si doping reduces the band edge states below the conduction band. According to density functional theory (DFT) calculations, Si, when it substitutes for Zn, acts as a carrier suppressor in the ZnON matrix.

## Introduction

Recently, zinc oxynitride (ZnON) thin-film transistors (TFTs) have attracted significant attention in next-generation, large size, and high-resolution display applications, because of their higher field-effect mobility^1–9^ than that of well-known amorphous indium-gallium-zinc oxide (a-IGZO) TFTs^1,10,11^. Previous studies on ZnON have shown that ZnON-TFTs with high field effect mobility can be obtained using RF sputtering system with Zn metal target under a mixed reactive gas atmosphere (of Ar, O_2_, and N_2_), which involves a simple and inexpensive process^2,3^. Optimization of the ZnON-TFT properties is normally performed by controlling the nitrogen (N) to oxygen (O) anion ratio^5,6^, or thermal annealing^7–9^. As a result, the ZnON-TFT obtains sufficiently low leakage current levels as well as high field effect mobility. It has been reported that the nitrogen vacant sites (V_N_) act as the major source of free electrons and carrier traps^3,12–14^ which may degrade the ZnON-TFT properties under bias stress. However, these previous studies have mainly focused on the field effect mobility on the device characteristics, and did not show enhanced bias stability. Therefore, the bias stability of the ZnON TFTs has yet to be fully clarified. Some previous studies have shown that the Si atoms could suppress oxygen deficiency^15–17^. Silicon oxide (Si-O, 798 kJ/mol)^18^ has high oxygen bond dissociation energy and high chemical stability, so it can improve TFT stability. Also, silicon nitride (Si-N, 439 kJ/mol)^18^ has higher bond dissociation energy than zinc nitride (Zn-N, 160 kJ/mol)^19^ and high chemical stability, so the N can be kept in stable states in the films. Due to these advantages, Si was selected. However, previous studies have mostly focused on the device characteristics, and have not shown detailed electronic structure such as conduction band edge states below the conduction band using x-ray absorption spectroscopy (XAS) and spectroscopic ellipsometry (SE) analysis and chemical bonding states such as Si peak using x-ray photoelectron spectroscopy (XPS) analysis. In this paper, we investigate the device performance of the ZnON and Si-ZnON TFTs, and find, via electronic structure, chemical bonding states, and first-principle calculations analysis, that silicon (Si) doping during the thin film growth by co-sputtering a SiO_2_ target with Zn metal target can more effectively improve the bias stability of ZnON TFTs.

## Results and Discussion

**Figure 1:**
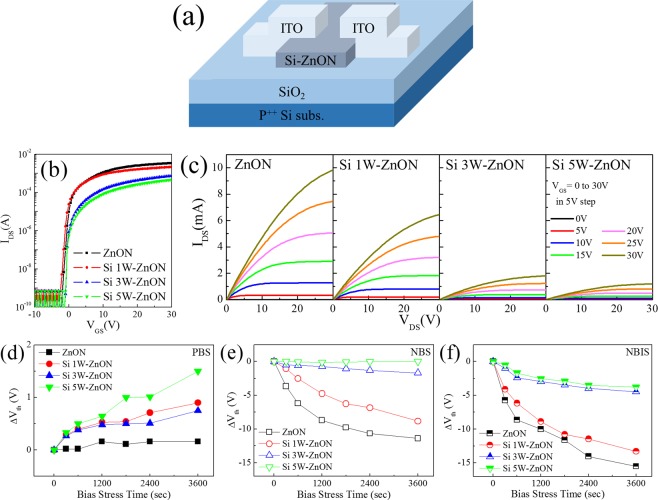
(**a**) Schematic of ZnON and Si-ZnON TFTs. (**b**) Transfer characteristics, and (**c**) output characteristics of the ZnON and Si-ZnON TFTs. The shift of threshold voltage under (**d**) PBS, (**e**) NBS, and (**f**) NBIS, of the ZnON and Si-ZnON TFTs.

Figure 1(a) shows a schematic of the TFT structure of ZnON and Si-doped ZnON. Figure 1(b) shows representative transfer characteristics of the TFTs with the Si-doped ZnON active layer deposited at SiO_2_ with RF powers of (0, 1, 3, and 5) W, which we indicate hereafter as ZnON, Si 1W-ZnON, Si 3W-ZnON, and Si 5W-ZnON, respectively. The field-effect mobility (μ_FE_) and threshold voltage (V_th_) in the saturation region (V_DS_ = 10 V) were calculated by fitting a straight line to the plot of the square root of I_DS_ versus V_GS_^20^. Table 1 shows the representative transfer parameters. As the Si doping power increased, the μ_FE_ of the Si-doped ZnON TFTs gradually decreased from 114.35 to 11.37 cm^2^/Vs for ZnON TFT to Si 5W- ZnON TFT. Si_3_N_4_ is well-known to be electrically insulating^21^, so the decrease in μ_FE_ is reasonable. Figure 1(c) shows representative output characteristics. As the Si doping power increased, the output curves of the Si-doped ZnON TFTs gradually decreased. This output curve trend is similar to the transfer curve behavior. Figure 1(d,e) show the positive bias stress (PBS) and negative bias stress (NBS) tests with a positive gate bias of 20 V and negative gate bias −20 V for 3,600 s, respectively, that we performed in order to evaluate the effect of Si doping on the device bias stability. In addition, Fig. 1(f) shows the negative bias illumination stress (NBIS) tests with a negative gate bias of −20 V and green light source with luminance of ~ 1,500 lx for 3,600 s that we performed. Figure S1 of the Supplementary Information (SI) shows that as the stress time of positive gate bias increases, ZnON and Si-doped ZnON TFTs undergo positive shifts in threshold voltage (ΔV_th_), without any significant degradations in device performance. The ΔV_th_ values under PBS are similar, but the ΔV_th_ under NBS and NBIS of the ZnON TFT is larger than that of the Si-doped ZnON TFTs. The NBIS test results are similar to the NBS test results, in that as the Si doping power increased, the ΔV_th_ gradually decreases. Si doping improved the stability of NBS by about 7 times (before Si doping, ΔV_th_ = −11.39 V; after Si doping, ΔV_th_ = −1.69 V). Si doping improved the stability of NBIS by about 4 times (before Si doping, ΔV_th_ = −15.53 V; after Si doping, ΔV_th_ = −3.78 V). As a result, we confirmed that Si doping improved the device stability. Previous reports identified two main mechanisms for the shift in V_th_. One of these is carrier trapping at the gate dielectric/semiconductor interface^22^, while the other is the creation of additional defect states in the deep-gap states at, or near, the gate dielectric/semiconductor interface^23^. The charge trapping affected by the defect near the gate dielectric/semiconductor interface is considered to be the main degradation mechanism of the ΔV_th_, due to the subthreshold swing (SS) value not showing significant variations. The V_N_ is ionized by holes that accumulate near the gate dielectric/semiconductor interface under NBS, and that release electrons^24,25^. The negative shift of V_th_ under NBS is related to the V_N_ migration toward the gate dielectric/semiconductor interface^26^. The NBIS degradation mechanism is similar to that of the NBS degradation, except for the effect of the green light. Under NBIS, photon radiation is likely to affect the ionization of V_N_. Therefore, the negative V_th_ shift value under NBIS is larger, and appears earlier than under NBS. Meanwhile, the remarkable decrease in the ΔV_th_ of the Si-ZnON TFTs under NBS and NBIS is related to the reduction of defects related to V_N_, which may affect free electron generation. On the other hand, the positive shift of V_th_ under PBS was mostly due to the negative charge trapping in the semiconductor and/or gate dielectric/semiconductor interface^27^. However, the difference of negative charge trapping in the gate dielectric/semiconductor interface between ZnON and Si-ZnON TFTs under PBS is negligible, and the changes of the positive ΔV_th_ did not show significant variations, compared with the changes of negative ΔV_th_ under NBS. These results indicate that Si significantly suppresses the free electron generation under NBS, while it has very little effect on the negative charge trapping under PBS. The passivation of such defects by Si doping is suggested to improve the NBS stability of the device based on Si-doped ZnON, as compared to that based on pure ZnON.

**Table 1:** Representative transfer parameters of the ZnON and Si-doped ZnON TFTs.

Sample	V_th_ [V]	μ_sat_ [cm^2^/Vs]	S.S. [V/decade]	I_ON_/I_OFF_
ZnON	−1.09	114.35	0.37	3.38 × 10^7^
Si 1W-ZnON	−1.56	94.95	0.38	2.12 × 10^7^
Si 3W-ZnON	−0.51	19.70	0.20	7.32 × 10^6^
Si 5W-ZnON	−0.51	11.37	0.22	4.62 × 10^6^

**Figure 2:**
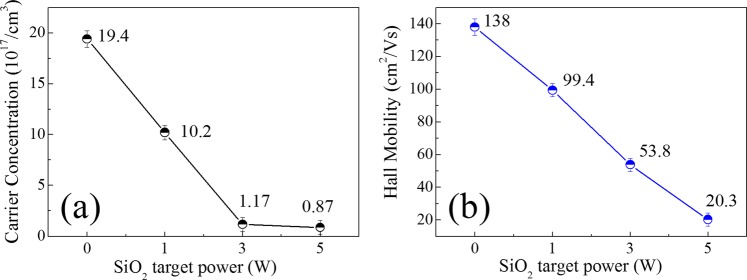
(**a**) Carrier concentration, and (**b**) Hall mobility, of the ZnON and Si-ZnON thin films.

Figure 2(a,b) show the carrier concentration and Hall mobility of ZnON and Si-ZnON thin films, respectively, that were evaluated by Hall measurements. Si doping dramatically reduced the carrier concentration and Hall mobility values. This result is reasonable because Si_3_N_4_ is well-known to be electrically insulating and these effects are similar to the tendency of decrease of the μ_FE_. For further analyses, the Si 3W-ZnON film was selected as the optimum condition.

**Figure 3:**
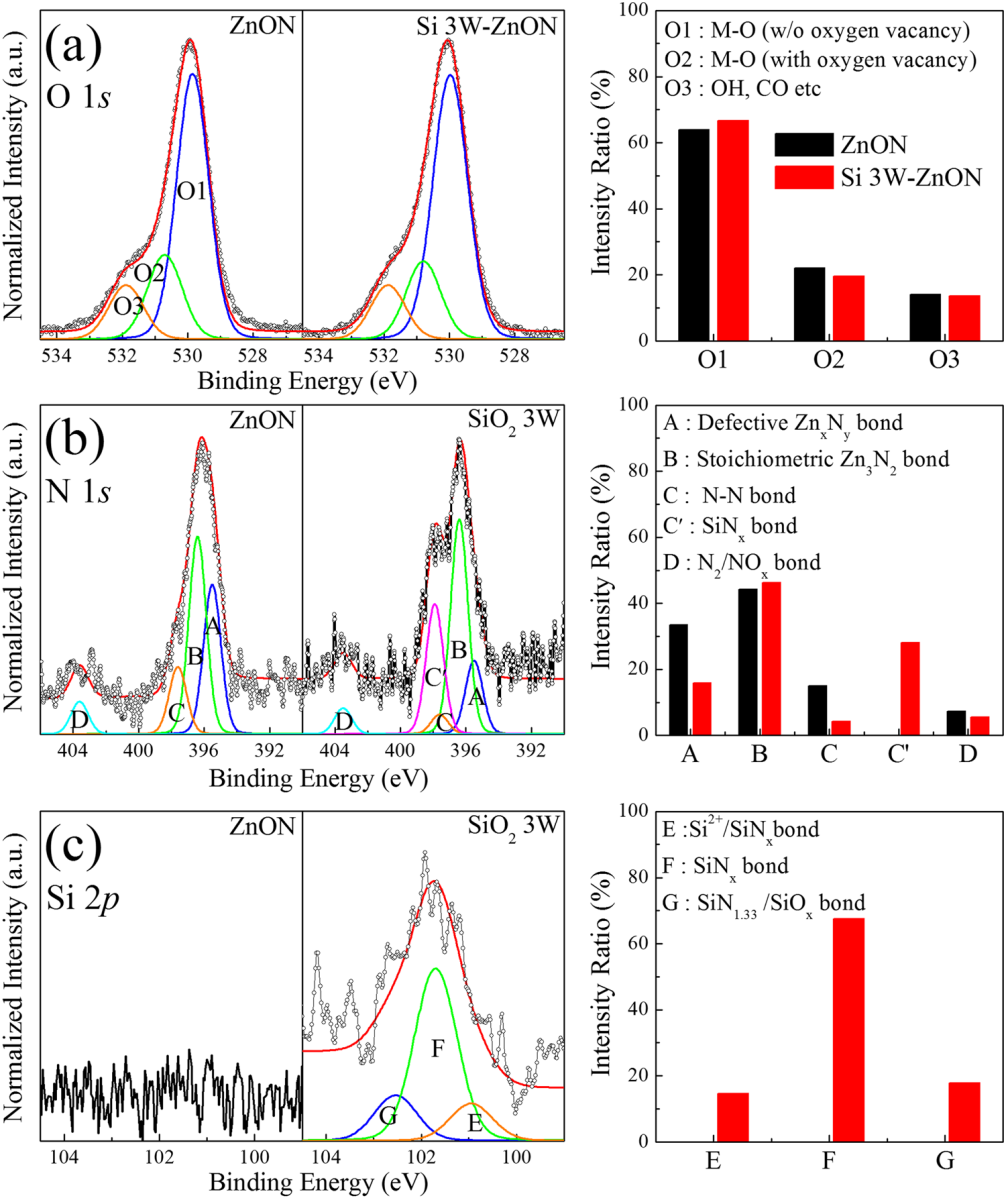
Deconvoluted XPS (**a**) O 1s, (**b**) N 1s, and (**c**) Si 2p spectra and relative intensity ratio, of the ZnON and Si 3W-ZnON thin films.

The Si doping concentrations, as well as changes in the chemical bonding states of ZnON and Si 3W-ZnON thin films, were examined through XPS analyses. The XPS spectra were measured after eliminating the surface contamination caused by adsorbed OH, C, H_2_O, etc., and using Ar^+^ ions at 500 eV, so as to minimize the preferred sputtering of light elements. Generally, all element spectra are calibrated by using the standard binding energy of carbon, which is of 284.5 eV. For this reason, we intentionally left the carbon. Based on previous studies^20,28^, Tables 2 and 3 summarize the estimated chemical composition and positions of the sub-peaks originating from specific bonds of the ZnON and Si 3W-ZnON layers, respectively. Figure 3(a) shows the O 1s spectra in XPS of the pure ZnON and Si 3W-ZnON films. The O 1s spectra were normalized and de-convoluted with three different Gaussian peaks, for detailed chemical bonding states of oxygen, which are located as low energy O1, middle energy O2, and high energy O3, respectively. The low binding energy (O1) to high binding energy (O3) peaks represent the metal-oxide (M-O) in ZnO lattices, the oxygen-deficient state within ZnO lattice, and the chemisorbed or dissociated oxygen states or OH− impurities, respectively^20,29^. Si doping slightly increased the relative areas of the M-O bonding states (O1), while it slightly decreased those of the oxygen-deficient bonding states (O2). Si doping decreased the oxygen vacancies related to the O2, which contributed to the decrease in carrier concentration^30^. However this effect did not significantly affect the carrier concentration drop. Figure 3(b) shows the XPS N 1 s peak spectra of the pure ZnON and Si 3W-ZnON layers. The lowest energy sub-peak originates from N atoms in nonstoichiometric Zn_x_N_y_ (Peak A, including V_N_), while peak B arises from the N atoms in stoichiometric Zn_3_N_2_. The C and C′ peaks are N-N and SiN_x_ bonding states, respectively. The highest energy sub-peak represents mostly N_2_ or NO molecules states^12,30,31^. The important changes are the significant decreases of nonstoichiometric Zn_x_N_y_ and N-N bonds closely related to the carrier generation, and the increases of stoichiometric Zn_3_N_2_ and SiN_x_ bonds with Si doping. These results may be interpreted as being due to the passivation of vacant N sites by Si that contribute to the sub-peak originating from nonstoichiometric Zn_x_N_y_. Consequently, it can be seen that the carrier concentration drop is related to the decrease of nitrogen vacancy. Figure 3(c) shows the XPS Si 2p peak spectra of the pure ZnON and Si 3W-ZnON layers. The three representatively assigned peaks from low binding energy are related to the Si^2+^, nonstoichiometric SiN_x_, and the SiN_1.33_ or SiO_x_ bonding states, respectively^30,32,33^.

**Table 2:** Chemical compositions of ZnON and Si 3W-ZnON film as deduced from XPS peak areas.

Sample	C	N	O	Si	Zn
	Composition (%)				
ZnON	5.3	4.2	42.3	—	48.2
Si 3W-ZnON	5.5	2.3	43.8	0.9	47.5

**Table 3:** Summary of XPS peak position with assignment of featured sub-peaks for the O 1 s, N 1 s, and Si 2p spectra.

Index	Chemical states	ZnON Position (eV)	Si 3W-ZnON Position (eV)	ZnON (%)	Si 3W-ZnON (%)
O1	Zn-O	529.85	529.98	63.84	66.66
O2	Oxygen deficient state	530.70	530.80	22.04	19.68
O3	CO, OH etc	531.88	531.88	14.13	13.66
A	Defective Zn_x_N_y_	395.50	395.50	33.46	15.85
B	Stoichiometric Zn_3_N_2_ bond	396.40	396.40	44.23	46.29
C	N-N bond	397.60	397.60	15.06	4.21
C′	SiN_x_ bond	—	397.90	—	28.06
D	NO_2_ bond	403.60	403.50	7.25	5.59
E	Si^2+^/SiN_x_bond	—	101.00	—	14.63
F	SiN_x_ bond	—	101.66	—	67.52
G	SiN_1.33_/SiO_x_bond	—	102.27	—	17.86

**Figure 4:**
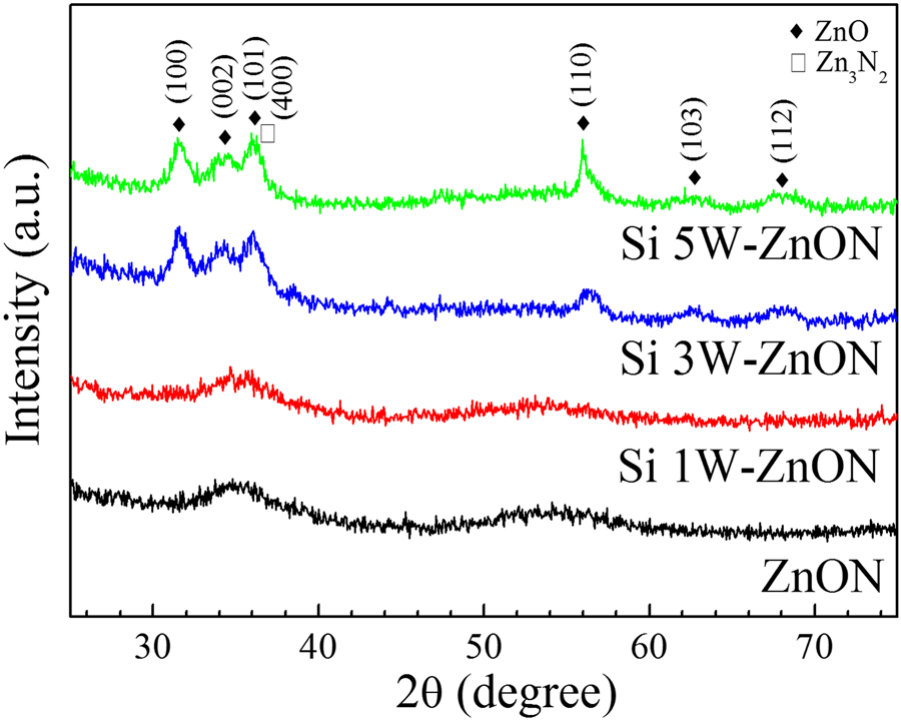
Grazing incidence angle x-ray diffraction patterns of the ZnON and Si-doped ZnON films.

In order to consider the physical structure of the ZnON and Si-doped ZnON thin films, we measured grazing incident angle x-ray diffraction (GIAXRD, Rigaku). Figure 4 shows the XRD patterns of the ZnON and Si-doped ZnON thin films that were obtained from 25° to 75° by using the fixed incident beam angle (ω, 1°) during 2θ. The XRD patterns of the ZnON and Si 1W-ZnON thin films exhibit amorphous structures; whereas, the Si 3 W and Si 5W-ZnON thin films exhibit six peaks, which correspond to the (100), (002), (101), (110), (103), and (112) peaks of hexagonal ZnO, and the peak positions overlap with the (400) peak of cubic Zn_3_N_2_^2,34^. These results are correlated to the increase of the ZnO bond and the stoichiometric Zn_3_N_2_ bond in Si 3W-ZnON film by XPS, as shown in Fig. 3. We believe that the four phases of Zn_3_N_2_, ZnO, amorphous ZnON (a-ZnON) and nano-crystallites ZnON (nc-ZnON) co-exist in Si 3 W and Si 5W-ZnON thin films. In order to elucidate the improvement of the device stability of the Si 3W-ZnON TFTs in terms of the electronic structures, we evaluated the conduction band and band edge state of the ZnON and Si 3W-ZnON films.

**Figure 5:**
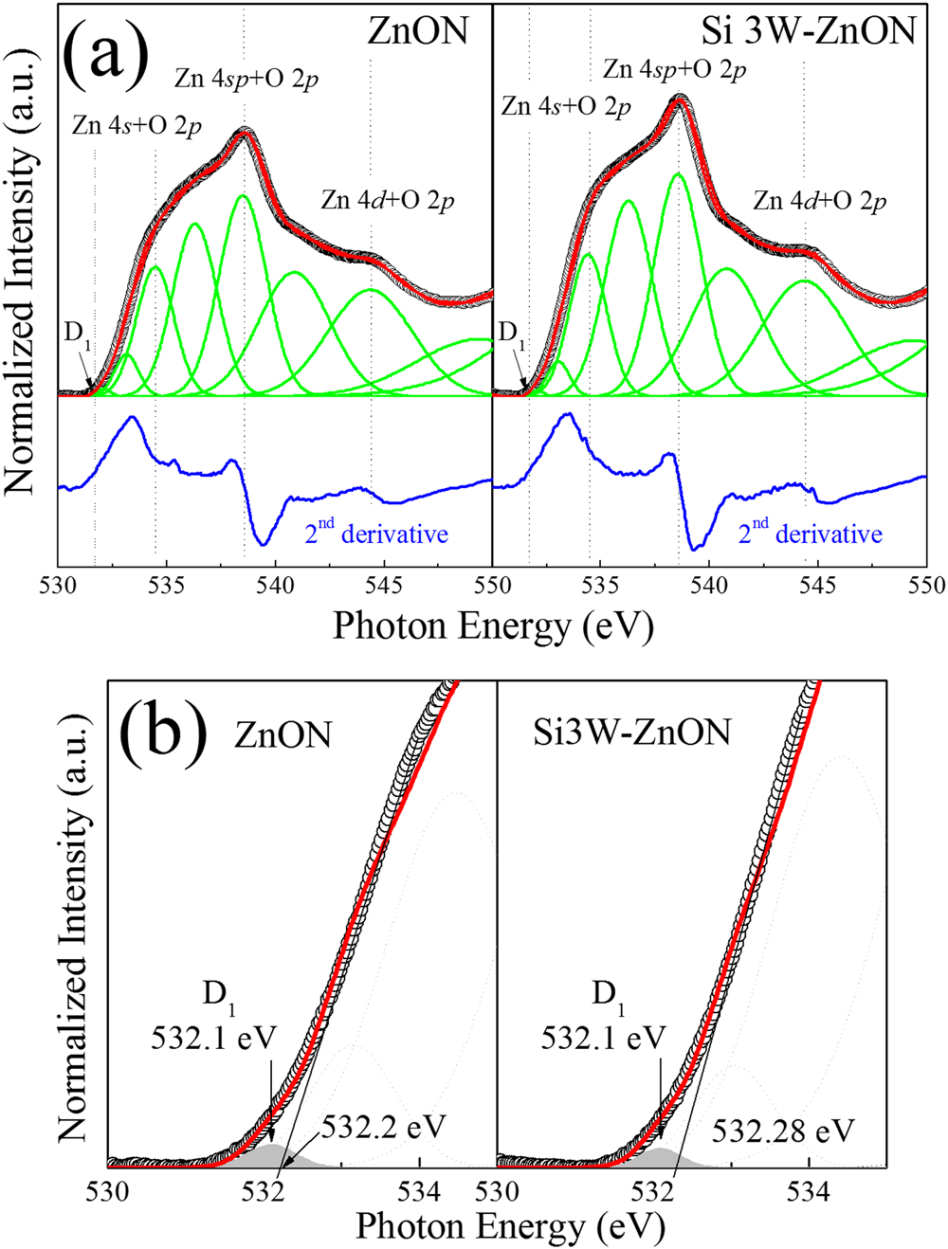
(**a**) Deconvoluted O K-edge XAS spectra, and (**b**) enlargement of the XAS spectra for band edge states below the conduction band, of the ZnON and Si 3W-ZnON thin films.

Figure 5(a) shows the XAS spectra of the normalized O K-edge, which provides more information over a wider conduction band, as well as the unoccupied hybridized states of the ZnON and Si 3W-ZnON films. The O K-edge spectra were obtained by deducting the x-ray background and scaling the post-edge levels to a uniform value. Following normalization, the relative intensity can explain the transition of an electron from the O 1s orbital to the unoccupied zinc 4s and 4sp molecular orbitals of ZnON based on symmetrically determined models, and the energy levels of the molecular orbital states can be obtained by the second derivative of the O K-edge spectra^35^. The band edge states D_1_ below the conduction band edge were detected from the second-derivative spectra. For further detailed analysis of the band edge states below the conduction band, Gaussian fits were performed. Figure 5(b) shows XAS O K-edge spectra over a narrow energy region below the conduction band for ZnON and Si 3W-ZnON films. The band edge states D_1_ of ZnON and Si 3W-ZnON films are 532.1 eV, and the conduction band positions of ZnON and Si 3W-ZnON films are 532.2 eV and 532.28 eV, respectively. The defect states D_1_ are close to the conduction band edge, which is located about 0.1 to 0.2 eV (for ZnON to Si 3W-ZnON, respectively) below the conduction band. The relative areas of D_1_ slightly decreased with Si doping. Previous studies on metal oxides have indicated some correlation between band-edge states and electrical properties such as carrier concentration and mobility^36,37^. Generally, the defect states below the conduction band edge might be divided into two states. One is the shallow band edge states close to the conduction band edge^38^. This relative defect state reduction below the conduction band edge is related to the decrease of the free electron by the generation of oxygen vacancies. The other is the deep band edge state far from the conduction band^39^. The degradation of carrier mobility is related to the deep band edge states, which are unoccupied states. In this state, the charge trapping and charge scattering are increased under carrier transport due to energy levels. In this paper, we obtained the defect states (D_1_) close to the conduction band edge, which is located about 0.1 to 0.2 eV (for ZnON to Si 3W-ZnON, respectively), below the conduction band. This defect state D_1_ is similar to the shallow band edge state type shown in some previous reports. Therefore, it seems that the reduction of the relative defect state is related to the decrease of the carrier concentration by Si doping. This tendency is similar to the decrease of oxygen vacancy (O2) in Fig. 3(a). However, it seems that these results will not have that much impact on the decrease of carrier concentration. The decrease of carrier concentration seems likely to be strongly influenced by the reduction of the Zn_x_N_y_ and N-N bond related to the V_N_, as shown in Fig. 3, rather than the decrease of oxygen vacancy.

**Figure 6:**
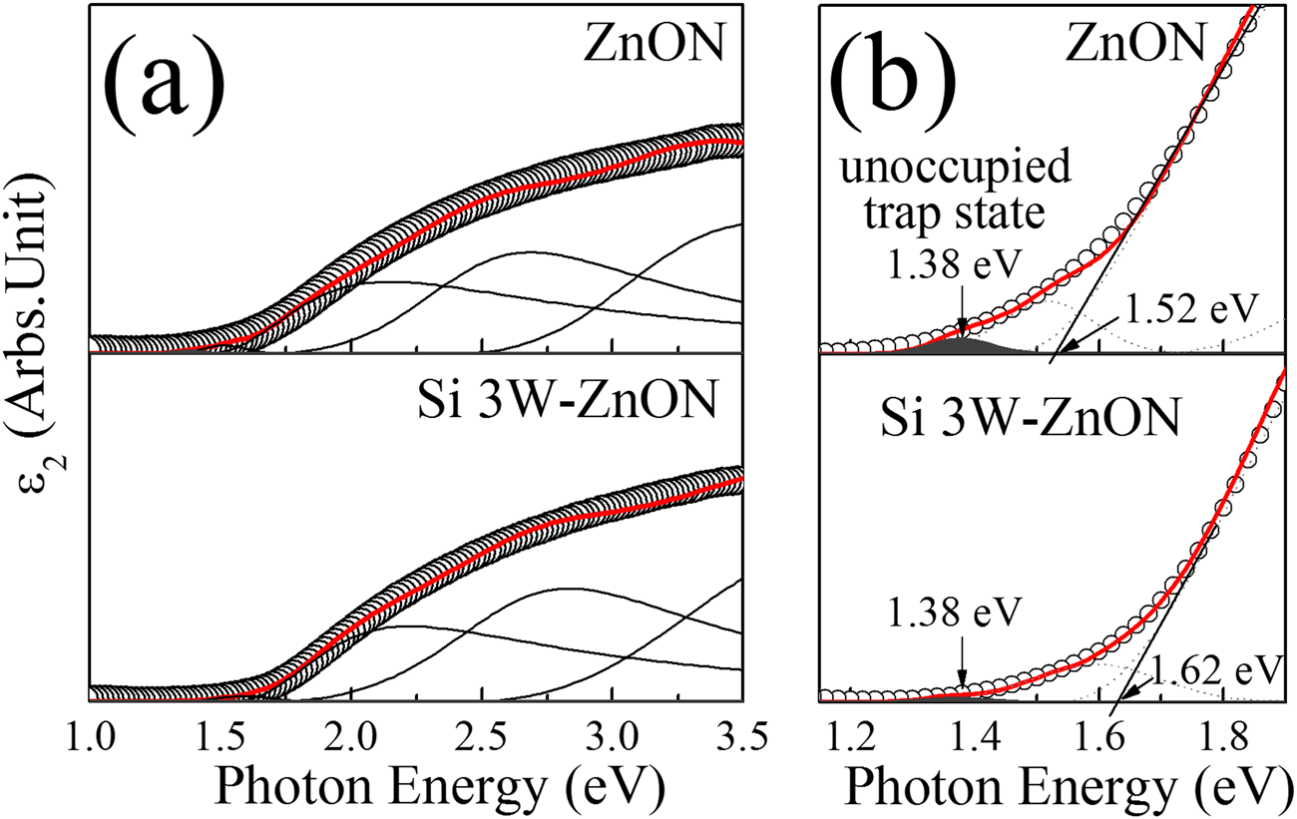
(**a**) Imaginary part of the dielectric function (ε2) spectra, and (**b**) enlargements of the ε2 spectra below the conduction band, of the ZnON and Si 3W-ZnON thin films.

Figure 6(a) shows the imaginary parts of the dielectric function (ε_2_) spectra that were obtained by spectroscopic ellipsometry (SE) using rotating analyser system with the incident angles of 65°, 70°, and 75° in the energy range 1 to 5 eV. These spectra were extracted from a simple four-phase model comprising a Si substrate, thermal SiO_2_ layer, ZnON layer, and an ambient layer. Further detailed and quantitative analysis of the band edge states and unoccupied trap states within the forbidden gap was performed using Gaussian function fitting^40^. The band gap of the Si 3W-ZnON films lies upward of ~ 0.1 eV relative to ZnON. Figure 6(b) shows the enlargement of the ε_2_ spectra for the band edge states below the conduction band, which represents the unoccupied trap state within the forbidden gap for the ZnON and Si 3W-ZnON films. The unoccupied trap state position lies close to the conduction band edge, which is located about 0.14 to 0.24 eV (ZnON to Si 3W-ZnON, respectively) below the conduction band. Si doping decreased the relative band edge state area, which results are consistent with those of previous XAS analyses.

**Figure 7:**
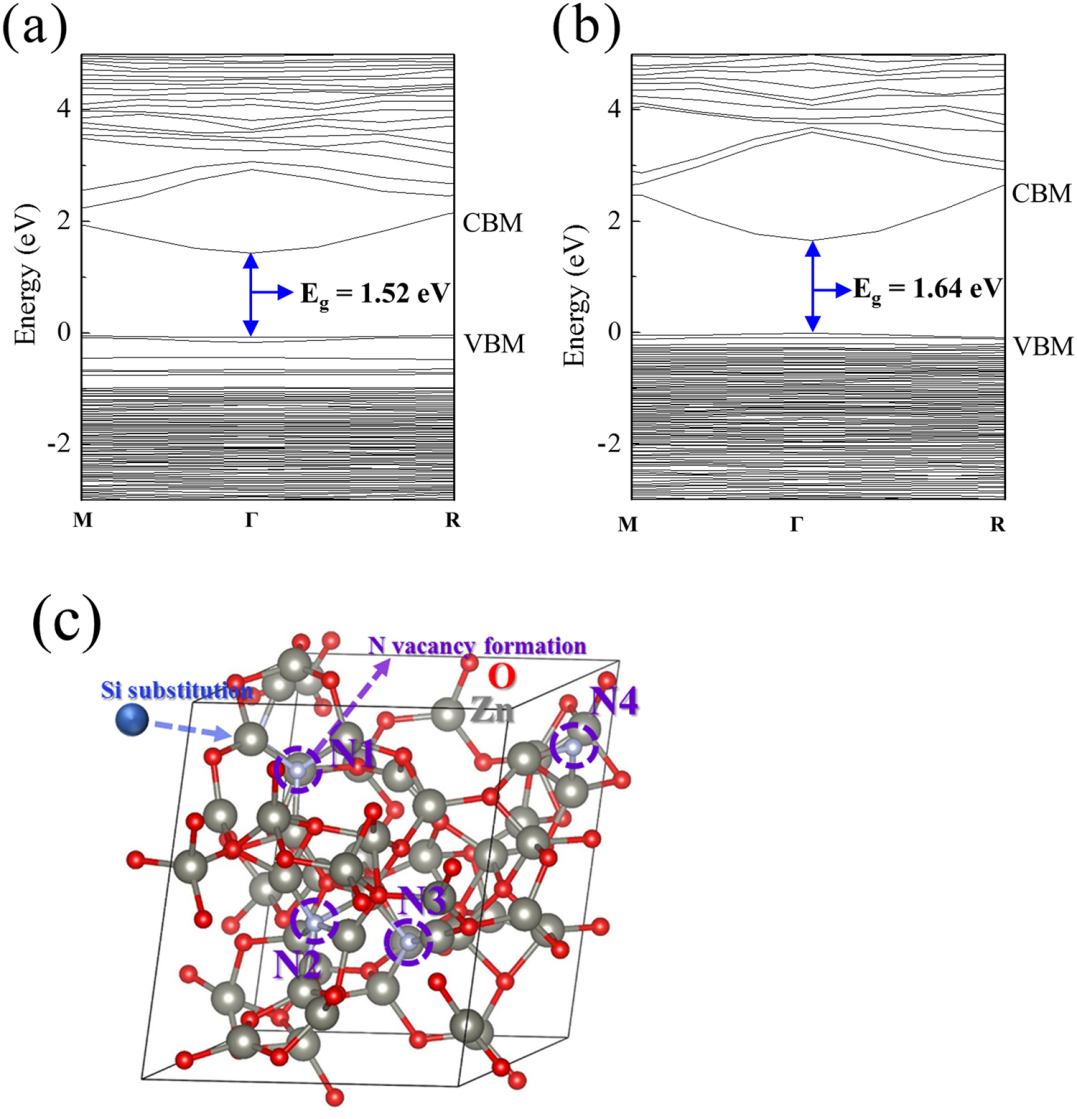
Calculated band structures of (**a**) ZnON, and (**b**) Si 3W-ZnON; and (**c**) schematic of the Si-doped ZnON.

In order to study the effects of Si doping in ZnON, MD simulations were performed to obtain amorphous ZnON supercells, and DFT calculations were adopted to generate the atomic structures of ZnON containing doped Si and various vacancies. Table 4 and Fig. 7(a,b) show the elemental ratio of amorphous ZnON and Si 3W-ZnON structures, and the calculated band gap energy of amorphous ZnON and Si 3W-ZnON by MD simulations, respectively, where the ratio and band gap energy were almost the same as the experimental data (Table 2 and Fig. 6(b)). Figure 7(c) shows N atoms in amorphous ZnON, which are labelled from N1 to N4, and Si substitution for Zn was carried out. The vacancy formation energies in ZnON and Si 3W-ZnON, E_form_ are calculated as follows:

1 $${{\rm{E}}}_{{\rm{form}}}={\rm{E}}({\rm{vacancy}})\,-\,{\rm{E}}({\rm{pure}})+{\mu }_{{\rm{element}}}$$

where E(vacancy) is the energy of ZnON or Si 3W-ZnON with certain vacancy such as N, O, Si, and Zn, while E(pure) corresponds to the energy of ZnON or Si 3W-ZnON without any vacancy. µ_element_ represents the chemical potential of elements, which was calculated by the energy of cation or the half energy of anion molecule^41^. E_form_(V_N_) was calculated by removing each N atom site (N1–N4) in ZnON or Si 3W-ZnON. Similarly, the O, Zn and Si vacancies were calculated by removing each atom.

**Table 4:** Atomic ratio of simulated ZnON and Si 3W-ZnON structures.

Structure	N	O	Si	Zn
	Atomic ratio (%)			
ZnON	4.5	44.4	0	51.1
Si 3W-ZnON	2.3	47.8	1.1	48.8

Table 5 shows the vacancy formation energies of each atom site in ZnON and Si 3W-ZnON structures. The calculation results indicate that the formation energies of N vacancies (V_N_) in the pure amorphous ZnON matrix were negative, or were almost zero values (E_form_ = −0.312 ± 0.255 eV), which means that V_N_s are easily formed and could act as defect states. However, when Si substitutes for Zn near each N atom, the formation energies are significantly increased (E_form_ = 1.516 ± 0.802 eV) from pure ZnON. Moreover, the formation energy of O vacancy (V_O_) is increased for the O near substituted Si, and the substituted Si had much higher vacancy formation energy than Zn vacancies (V_Zn_)_._ These results are consistent with the experimental data, i.e. the suppression of anion vacancy formation by Si doping in amorphous ZnON.

**Table 5:** Vacancy formation energies of each N site in ZnON and Si 3W-ZnON structures.

Type	ZnON				Si 3W-ZnON			
	Site 1	Site 2	Site 3	Site 4	Site 1	Site 2	Site 3	Site 4
	Formation energies (eV)							
V_N_	−0.567	−0.188	0.057	−0.442	2.318	1.549	0.714	1.581
V_O_	3.079	3.446	3.093	3.027	4.788	4.952	4.506	4.491
V_Zn_	2.879	3.414	3.093	3.250	N/A	N/A	N/A	N/A
V_Si_	N/A	N/A	N/A	N/A	6.049	5.552	3.801	5.866

**Figure 8:**
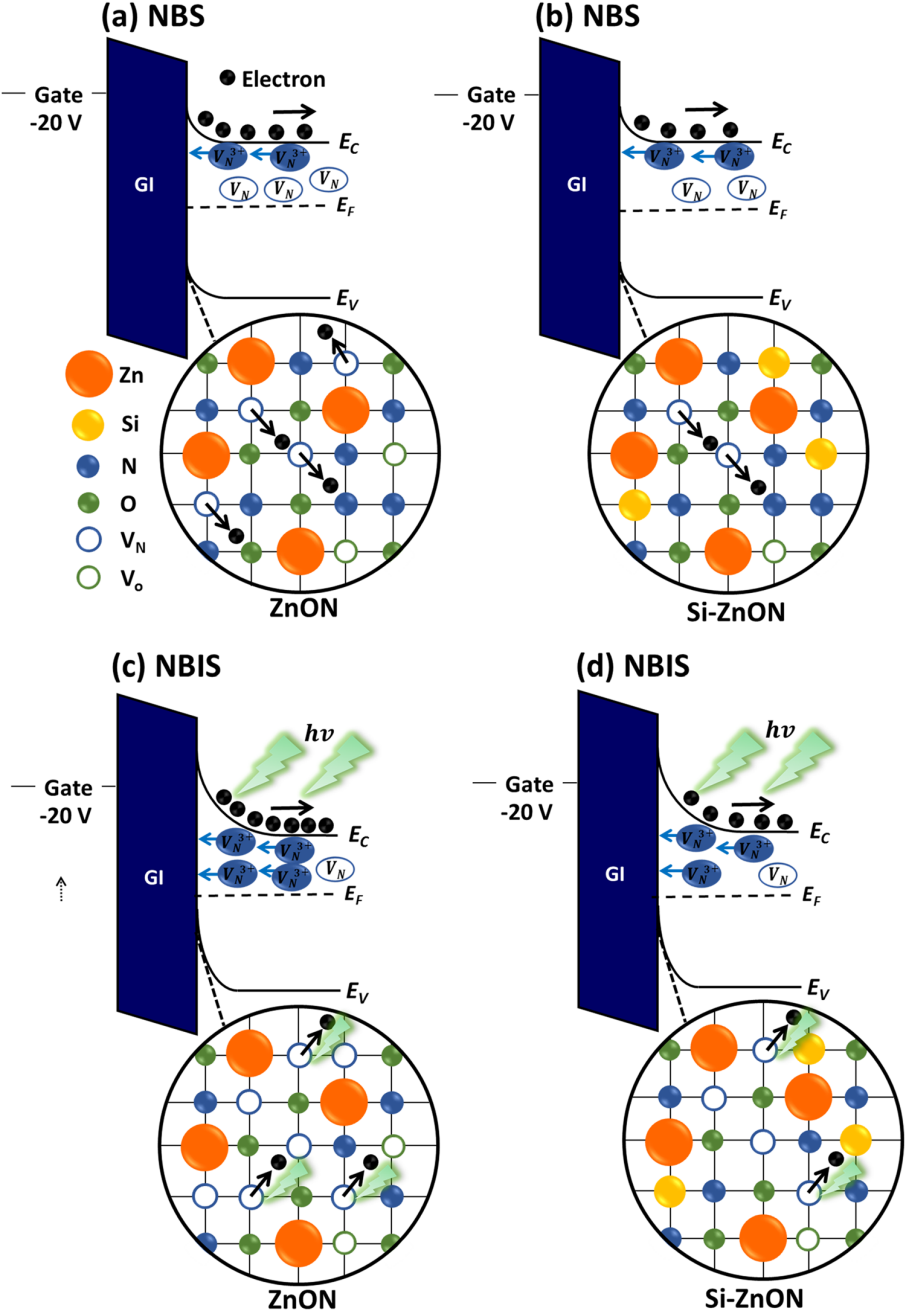
Schematic degradation mechanism of (**a**) ZnON TFT, and (**b**) Si 3W-ZnON TFT under NBS; (**c**) ZnON TFT, and (**d**) Si 3W-ZnON TFT under NBIS.

Figure 8(a,b) show schematics of the degradation mechanism under NBS with band bending states on the gate dielectric/channel interface of ZnON and Si 3W-ZnON TFTs, respectively. Figure 8(c,d) show the degradation mechanism of ZnON and Si 3W-ZnON TFTs respectively, under NBIS. These band bending states under NBS and NBIS will be partially occupied by V_N_^3+^, and cause the Fermi energy (E_F_) level to shift by higher than half value of the band gap by n-type semiconductor characteristics. As a result, these are usually electron donor states, as they will tend to increase electron occupation of the conduction band. Meanwhile, these states are closely associated with degradation of the device stability.

To be more specific, when a negative bias is applied at the gate of the ZnON TFT, the E_F_ shifts towards the middle of the band gap, and V_N_^3+^ migrates more closely toward the gate dielectric/channel interface. Furthermore, the amount of electron and electron trap sites increases, due to the released electrons from the weakly bonded unstable defect states (V_N_ related to ZnO_x_N_y_, and/or Zn_x_N_y_), thereby deteriorating the stability of the device as shown in Fig. 8(a,b). In the case of NBIS, the greater amount of V_N_^3+^ accumulated at the gate dielectric/channel interface will increase by photon radiation, as shown in Fig. 8(c,d). However, in the case of Si 3W-ZnON TFT, the unstable N defect states and released electrons were decreased, due to the Si likely suppressing the V_N_ sites. Considering the previous electrical data of device bias stability, chemical bonding states, band edge states below the conduction band, and DFT simulations shown respectively from Figs 1–7, device instability is strongly correlated with V_N_, and Si doping could be the plausible origin of the enhanced device bias instability. Si doping decreased the V_N_ defects, which can enhance the device bias stability in Si 3W-ZnON by suppressing free electron generation.

## Conclusion

In conclusion, we evaluated the electrical properties of Si-doped ZnON thin film transistor and interpreted their origins using experimental analysis and theoretical calculation. Si 3 W (~1%)-doped ZnON TFTs showed a saturation mobility of 19.70 cm^2^/Vs as well as dramatic improvements in the threshold voltage shift for NBS within 1.69 V. Doped Si substitutes for zinc sites and induces increases in stoichiometric Zn_3_N_2_ bonds and decreases in defective Zn_x_N_y_ bonds. DFT calculations also suggest that Si is likely to suppress V_N_ formation. Therefore, the improvement of bias stability in Si-doped ZnON TFT originates from the strong suppression of V_N_s_,_ and the small number of electron-trap sites.

## Methods

### Fabrication of the Si-ZnON TFTs

First, 40nm-thick ZnON and Si-doped ZnON layers were grown on highly doped *p*-type Si substrates with a thermally grown 100 nm-thick SiO_2_ dielectric layer by co-sputtering Zn and SiO_2_ targets. The power exerted on the Zn metal target and on the SiO_2_ target was fixed at DC 100 W and varied, respectively. The reactive gas flow rate ratio was Ar:O_2_:N_2_ = 3:3.4:40 and the chamber pressure was 5 mTorr. Then, an indium tin oxide source/drain electrode was deposited and patterned using shadow masks. The fabricated TFTs had a bottom gate structure as well as a channel width (W) and length (L) of 800 and 200 μm, respectively. Finally, the ZnON and Si-doped ZnON TFTs were annealed at 300 °C for 1 h in air atmosphere, using a furnace system. The transfer characteristics and bias stability of the ZnON TFTs with respect to the Si doping power were measured at room temperature (RT), using a semiconductor parameter analyzer.

### Electrical and physical measurements

The transfer characteristics, output characteristics, and gate bias stability behaviors of the ZnON TFT and Si-ZnON TFT devices were measured at RT using the Keithley SCS-4200 semiconductor-parameter analyzer. The electrical properties, such as carrier concentration and Hall mobility, of ZnON and Si-ZnON thin films were measured at RT using the Hall measurement system with 0.56-Tesla permanent magnet. During the electrical measurements, the drain-to-source voltage (*V*_*DS*_) was fixed at 10 V, and the drain-to-source current (*I*_*DS*_) was measured in gate-to-source voltage (*V*_*GS*_) from −30 to 30 V. GIAXRD with incident beam angle 1°, was performed in order to obtain the physical structure of the ZnON and Si-doped ZnON thin films, using Cu Kα radiation (Rigaku).

### Electronic-structure measurements

The changes of the chemical bonding states depending on the Si doping were examined by XPS using a monochromatic Al Kα source with a pass energy of 29.35 eV. In addition, the electronic structure and band edge states below the conduction band of the Si-doped ZnON films were examined by XAS through the total electron yield mode at the 10D beamline of the Pohang accelerator laboratory in Korea. The electronic structures, regarding changes in the absorption coefficient and band alignment, were investigated by SE and XPS. The SE measurements were performed using a rotating-analyzer system with auto retarder.

### Theoretical calculations

The Vienna Ab-initio Simulation Package (VASP) was performed for the first-principles calculations employing a plane wave basis set with a kinetic energy cutoff of 400 eV, within the projector augmented wave (PAW) pseudopotentials^42,43^. Melt-and Quench Molecular Dynamics (MD) simulations were used to make amorphous ZnON structures based on the elemental ratio of ZnON from XPS data^24^. Density Functional Theory (DFT) simulations were carried out to calculate the formation energies of nitrogen vacancies (V_N_) in amorphous ZnON structure. All calculations used the generalized gradient approximation (GGA) method^44^, and the Brillouin zone was sampled with 1 × 1 × 1 (MD) and 2 × 2 × 2 (DFT) **k**-points mesh. The HSE06 hybrid-functional with 20% of the HF exchange energy was modified, in order to fit the experimental bandgap of the ZnON and Si-doped ZnON structures.

## Supplementary information

Supplementary Information.
