# Supplementary material for: Significant enhancement of the bias stability of Zn-O-N thin-film transistors via Si doping
Source: Sci Rep. 2020 Jan 20;10:719. doi: 10.1038/s41598-020-57642-2 (PMC6970993; doi:10.1038/s41598-020-57642-2)
Supplement: Supplementary file 1 — Supplementary Information. [file 41598_2020_57642_MOESM1_ESM.doc]

**Significant enhancement of the bias stability of Zn-O-N thin-film transistors via Si doping**

Aeran Song^1^, Hyun-Woo Park ^1^, Hyoung-Do Kim^2^, Hyun-Suk Kim^2,*^, Kwun-Bum Chung ^1^,*[[1]](#footnote-2)*

^1^Division of Physics and Semiconductor Science, Dongguk University, Seoul, 04620, Republic of Korea

^2^Department of Materials Science and Engineering, Chungnam National University, Daejeon 34134, Republic of Korea

***Supplementary figure S1****. Time evolution of the transfer curve under (a) PBS, (b) NBS, and (c) NBIS of the ZnON and Si-ZnON TFTs.*

*
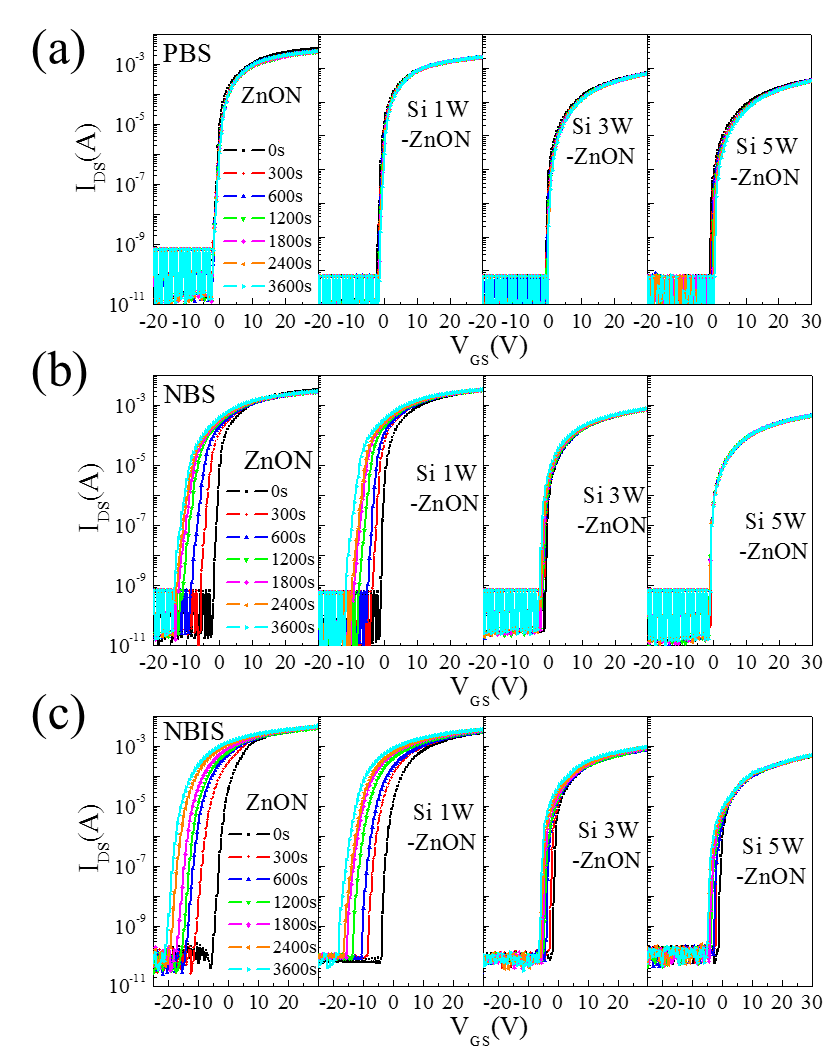
*

1. e-mail of corresponding Authors:

   khs3297@cnu.ac.kr (Hyun-Suk Kim), kbchung@dongguk.edu (Kwun-Bum Chung) [↑](#footnote-ref-2)
